# Cardiovascular Risk and Bone Mineral Density in Children with Familial Mediterranean Fever: The Role of Disease Severity and Genetic Mutations

**DOI:** 10.3390/ijms27135970

**Published:** 2026-07-03

**Authors:** Emrah Çığrı, Funda Çatan İnan, Sedat Gülten, Fethiye Yildiz, Mehmet Akif Bildirici, Hilmi Onur Kabukçu, Eren Yildiz, Metin Asileren, Ayşe Ece Gökkaya, Elif Aksu, Eren Er

**Affiliations:** 1 Department of Pediatrics, Kastamonu University, Kastamonu 37150, Türkiye; fyildiz@kastamonu.edu.tr (F.Y.); eren70@gmail.com (E.Y.); 2 Biostatistics and Medical Informatics, Bilecik Şeyh Edebali University, Bilecik 11230, Türkiye; funda.catan@bilecik.edu.tr; 3 Medical Biochemistry, Kastamonu University, Kastamonu 37150, Türkiye; sgulten@kastamonu.edu.tr (S.G.); mabildirici@kastamonu.edu.tr (M.A.B.); 4 Department of Pediatrics, Private Kastamonu Anatolia Hospital, Kastamonu 37200, Türkiye; onurkabukcu89@gmail.com; 5 Department of Pediatrics, Kastamonu Training and Research Hospital, Kastamonu 37150, Türkiye; metinasileren@gmail.com (M.A.); ecegokkaya@hotmail.com (A.E.G.); drelfzgvn@gmail.com (E.A.); 6 Pediatric Endocrinology, Ege University, İzmir 35040, Türkiye; drerener1984@gmail.com

**Keywords:** child, cardiovascular risk, Familial Mediterranean Fever, bone mineral density

## Abstract

In the present study, cardiovascular risk indices (Atherogenic Index of Plasma [AIP], Atherogenic Coefficient [AC], Castelli Risk Index-1 [CRI-1], and Castelli Risk Index-2 [CRI-2]) and bone mineral density (BMD SDS) were evaluated in patients with Familial Mediterranean Fever (FMF), and the association of these parameters with disease severity and *MEFV* gene mutations was investigated. A total of 126 participants (96 FMF patients and 30 healthy controls) were included in the study. FMF patients were classified as mild, moderate, or severe according to the PRAS severity score. Cardiovascular risk indices, biochemical parameters of bone metabolism, and BMD SDS values were compared among the groups. The relationship between BMD SDS and atherogenic indices was assessed by Spearman correlation analysis. The results were also analyzed according to *MEFV* gene mutations. No significant difference was detected among the groups in terms of sex or age (*p* > 0.05). FMF patients were found to have substantially higher cardiovascular risk indices than the control group, and these indices increased in parallel with disease severity (*p* < 0.05). The BMD SDS value of the severe FMF group was significantly lower than that of the other groups (*p* = 0.025). No difference was detected among the groups with respect to bone metabolism parameters (*p* > 0.05). No significant correlation was observed between BMD SDS and cardiovascular risk indices (*p* > 0.05). *M694V* was the most frequently detected mutation, and the BMD SDS value was lower in patients carrying mutations other than *M694V* (*p* = 0.011). The increased cardiovascular risk in FMF patients is associated with disease severity. Severe disease is accompanied by a reduction in bone mineral density. The lack of an association between cardiovascular risk and bone mineral density suggests that these systems may be affected through different mechanisms. It is important that FMF patients are monitored with respect to cardiovascular and bone health.

## 1. Introduction

FMF is one of the most common autoinflammatory diseases of childhood; it is inherited in an autosomal recessive manner and is characterized by serositis and recurrent febrile attacks. The principal pathogenesis of the disease is related to overactivation of the pyrin inflammasome secondary to *MEFV* gene mutations and to the resulting chronic inflammatory response [1,2].

Although asymptomatic periods are present between attacks, inflammatory activity is thought to remain incompletely suppressed, and this may contribute to organ damage in the long term. Attack frequency varies from patient to patient and can range from once every three to four months to weekly or more frequent occurrences. Attack periods can substantially impair daily life and stand in clear contrast to the complete sense of well-being observed during attack-free intervals [3].

FMF is frequently encountered, particularly in populations originating from the Mediterranean basin such as Turks, Armenians, Jews, and Arabs [4]. Turkey is one of the countries in which the disease is most frequently observed, with a prevalence of approximately 1:1000 [5]; however, in one study, the prevalence was found to decrease to 6:10,000 in the northwestern regions [6].

Familial Mediterranean fever is characterized by persistent activation of innate immune pathways mediated by the pyrin inflammasome. Increased caspase-1 activity and subsequent release of pro-inflammatory cytokines, particularly interleukin (IL)-1β and IL-18, may contribute to chronic subclinical inflammation even during attack-free periods. Sustained inflammatory activity has been associated with endothelial dysfunction, increased arterial stiffness, and other early vascular alterations that may predispose patients to cardiovascular disease. In addition, chronic inflammation may adversely affect bone remodeling by disrupting the balance between bone formation and resorption, potentially leading to reduced bone mineral density. Therefore, inflammatory mechanisms associated with FMF may represent a common biological pathway linking cardiovascular risk and skeletal health impairment [1,5].

Although studies addressing cardiovascular risk markers and bone mineral density in children with FMF are available, most previous reports have evaluated these outcomes separately or in adult populations. In contrast, the present study uniquely investigates both cardiovascular risk indices and bone mineral density parameters simultaneously in a pediatric cohort, while also considering disease severity and *MEFV* gene mutation status. This integrated approach may provide a more comprehensive understanding of the interplay between inflammation, genetic background, and long-term disease-related complications in FMF.

## 2. Results

A total of 126 participants (51 [40.5%] boys and 75 [59.5%] girls) were included in the study, and the mean age was 9.22 ± 4.69 years. No significant difference was observed among the four groups in terms of age (*p* = 0.992) or sex (*p* = 0.870).

**Figure 1:**
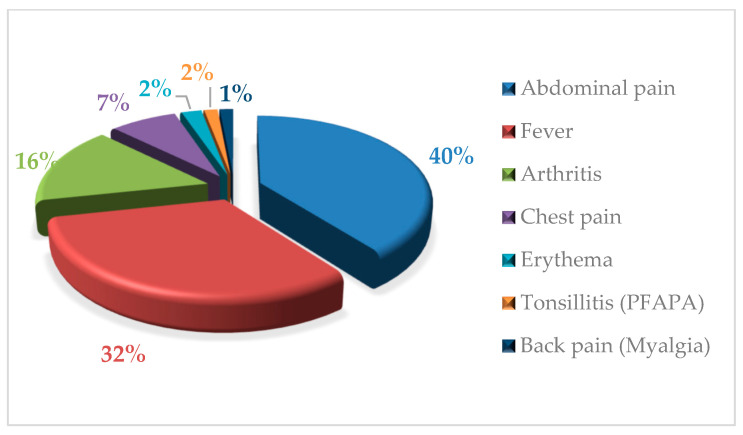
Distribution of clinical symptoms in patients with FMF.

**Figure 2:**
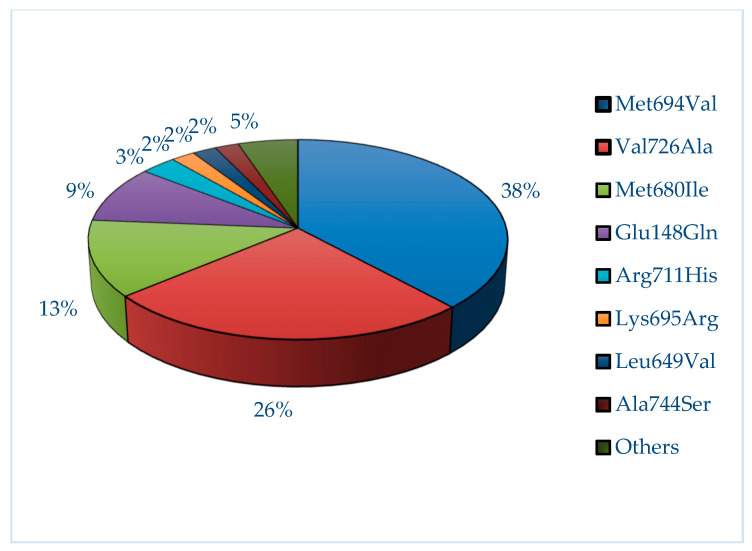
Distribution of *MEFV* gene mutations among FMF patients.

The distribution of clinical symptoms in patients with FMF is shown in Figure 1, and the distribution of *MEFV* gene mutations is shown in Figure 2. The symptoms shown in Figure 1 represent clinical manifestations recorded at the time of FMF diagnosis.

Post hoc analyses demonstrated significant pairwise differences among the groups with respect to the cardiovascular risk indices AIP, AC, CRI-1, and CRI-2 (*p* < 0.05). The cardiovascular risk indices of FMF patients were found to be significantly higher than those of the control group, and these indices were observed to rise significantly as disease severity increased (*p* < 0.001) (Table 1).

Since cardiovascular risk indices were derived from lipid profile parameters, actual lipid concentrations were reported for all groups (Table 2).

In post hoc comparisons, the BMD SDS value was found to be significantly lower in FMF patients in the severe group than in FMF patients with moderate and mild disease and in the control group (*p* = 0.025). No significant difference in BMD SDS was detected between FMF patients with moderate or mild disease and the control group (*p* > 0.05). The biochemical parameters related to bone metabolism, namely Ca, P, ALP, vitamin D, and PTH, were observed to be within normal ranges in all four groups, and no statistically significant difference was found among the groups for these parameters (*p* > 0.05) (Table 3).

In the Spearman correlation analysis, no significant correlation was detected between BMD SDS and the atherogenic indices AIP, AC, CRI-1, and CRI-2 (Table 4).

In comparisons between FMF patients with the *M694V* mutation and FMF patients with mutations other than *M694V*, no significant differences were found in the AIP, AC, CRI-1, or CRI-2 measurements (*p* > 0.05), whereas the BMD SDS value was found to be significantly lower in patients carrying mutations other than *M694V* than in those carrying the *M694V* mutation (*p* = 0.011) (Table 5).

## 3. Discussion

In the present study, cardiovascular risk indices in children and adolescents diagnosed with FMF were shown to be significantly higher than those in healthy controls, and they were observed to rise markedly as disease severity increased. In addition, bone mineral density was found to be significantly reduced in the severe disease group. However, no significant correlation was found between cardiovascular risk indices and BMD. In the genotype-based analysis, BMD SDS was determined to be lower in patients carrying mutations other than *M694V*.

Familial Mediterranean Fever is a chronic inflammatory disease caused by mutations in the *MEFV* gene, which encodes the pyrin protein that is thought to play a role in inflammatory pathways. These mutations may result in loss of pyrin function, leading to uncontrolled inflammation, which in turn may give rise to endothelial dysfunction, prothrombotic states, and functional alterations in the microcirculation, ultimately predisposing to atherosclerosis [7]. In addition, FMF patients have been shown in previous studies to have abnormal lipid profiles [8], and epidemiological studies have demonstrated that dyslipidemia on its own is sufficient to drive the atherosclerotic process even in the absence of other risk factors.

A growing body of evidence from recent pediatric and adult studies has demonstrated the utility of atherogenic indices as markers of atherosclerosis and cardiovascular disease, as well as their value in predicting clinical outcomes and prognosis [9,10,11,12,13]. Previous pediatric studies have demonstrated evidence of early vascular alterations in children with FMF, including increased carotid intima–media thickness and increased arterial stiffness, suggesting a predisposition to premature atherosclerosis [14,15]. In our study, in line with the literature, the cardiovascular risk indices were found to be significantly higher in FMF patients than in the healthy control group, and these indices were also observed to rise significantly as the severity of FMF increased. The observation that cardiovascular risk indices are higher in FMF patients than in healthy controls and become more pronounced with increasing disease severity suggests that these parameters could be used in clinical follow-up as early markers of subclinical atherosclerosis. However, despite being widely accepted indicators of cardiovascular risk, AIP, AC, CRI-1, and CRI-2 do not provide direct assessments of endothelial dysfunction, vascular damage, arterial stiffness, or the burden of atherosclerotic plaques. Therefore, the present findings should be interpreted as indicators of increased cardiovascular risk rather than evidence of established cardiovascular disease. Future studies incorporating vascular imaging modalities such as carotid intima–media thickness or endothelial function assessments may provide more direct evidence of cardiovascular involvement in FMF.

The most widely used method to assess bone mineral density (BMD) is dual-energy X-ray absorptiometry (DXA) [16]. Because peak bone mass is not attained until the second or third decade of life [17], comparing children’s BMD with that of young adults is not appropriate; for this reason, T-scores should not be used in the pediatric age group. Instead, calculation of the BMD Z-score, which represents the SD score relative to age-matched controls, is a more appropriate approach [18].

In FMF patients, inflammatory cytokines such as IL-1, IL-6, IL-17, and TNF-α stimulate osteoclasts, increase bone resorption, and inhibit osteogenesis, thereby reducing bone mineral density [19,20]. Previous studies have also reported that BMD and BMD Z-scores are significantly lower in children diagnosed with FMF than in control groups [21,22,23,24]. Similarly, adult studies have also reported that BMD Z-scores in FMF patients are significantly lower than those in controls [25,26]. On the other hand, in the studies conducted by Koşan et al. [27] and Atağ et al. [28] in children diagnosed with FMF, no significant association was found between disease severity score and BMD Z-score. In our study, although BMD SDS values were significantly lower in patients with more severe FMF, the observed differences were relatively modest and did not indicate overt skeletal pathology. According to current pediatric densitometry guidelines, none of the participants fulfilled the diagnostic criteria for pediatric osteoporosis, suggesting that the observed reductions in BMD may reflect subclinical effects of chronic inflammation rather than clinically manifest bone disease.

It has been proposed that, in chronic inflammatory diseases, atherosclerosis and osteoporosis may develop through shared inflammatory pathways [29]. Accordingly, a significant association between cardiovascular risk and bone mineral density might be expected. However, no significant correlation was detected between BMD SDS and atherogenic indices in our study. On this basis, it can be stated that, in FMF, the responses of the cardiovascular system and bone tissue to inflammation are regulated through different mechanisms.

*MEFV* gene mutations, which constitute the genetic basis of Familial Mediterranean Fever, can influence the disease phenotype and severity. In particular, the *M694V* mutation has been reported to be associated with a more severe clinical course through its predisposition to the development of amyloidosis [30,31]. Additional studies have also demonstrated that a more severe phenotype, with high fever, splenomegaly, and musculoskeletal manifestations, is generally associated with high-penetrance mutations such as *M694V* [32,33]. In another study, however, no difference was observed in disease severity scores between common mutations (*M694V, V726A*, and *M680I*) and rare mutations [34]. Likewise, in the study by Salah et al. [24], no significant association was detected between *MEFV* gene mutation and BMD Z-score in children with FMF. Siverekli et al. [20] also reported that no significant association was found between mutation type and BMD. In our study, bone mineral density (BMD) values were significantly lower in patients carrying *MEFV* mutations other than *M694V* compared with those carrying the *M694V* mutation. This finding should be interpreted cautiously, as BMD SDS values in the overall cohort were generally within the expected age-adjusted range, which limits the clinical implications of the observed difference. Although this result may indicate potential genotype-related differences in bone metabolism, its clinical significance remains uncertain and requires confirmation in larger, well-characterized cohorts. The non-*M694V* group is inherently heterogeneous, consisting of multiple *MEFV* variants with potentially distinct biological effects, which further complicates interpretation. Several unmeasured factors, including physical activity, nutritional status, disease duration, and cumulative inflammatory burden, may have contributed to the observed association. Therefore, this finding should be considered hypothesis-generating rather than definitive evidence of a mutation-specific effect on bone mineral density. Further multicenter studies with larger sample sizes and detailed genotypic stratification are warranted to validate these observations.

## 4. Materials and Methods

**Figure 3:**
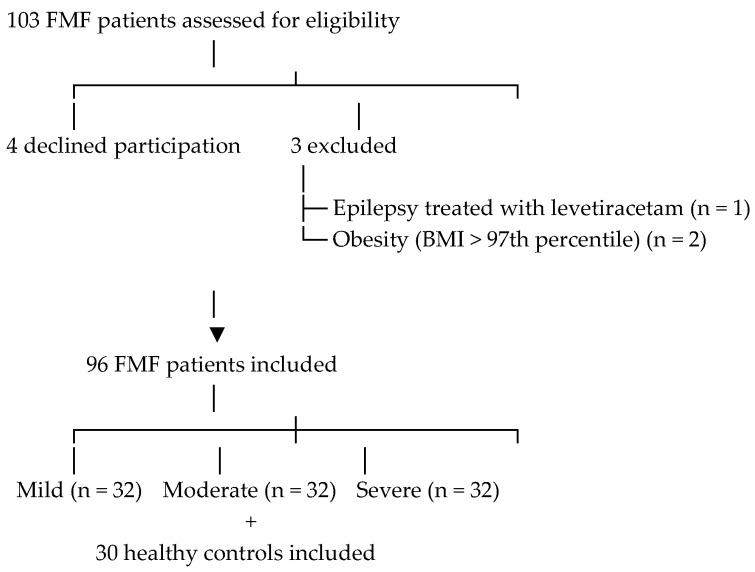
Participant recruitment and selection process.

This study was planned as a cross-sectional and comparative study conducted at the Pediatrics Clinic of Kastamonu Training and Research Hospital between January 2026 and May 2026. A total of 126 children and adolescents, comprising 96 FMF patients and 30 healthy controls, were enrolled in the study. The control group consisted of healthy children and adolescents without chronic diseases who were recruited during the same study period. Controls were selected to achieve an overall age and sex distribution comparable to that of the FMF cohort; however, individual matching was not performed. All FMF patients were receiving regular colchicine treatment according to standard clinical practice. The prescribed colchicine dose was 0.5 mg/day for children younger than 5 years, 1.0 mg/day for those aged 5–10 years, and 1.0–1.5 mg/day for patients older than 10 years. All patients had been receiving colchicine therapy for at least 6 months before study enrollment, and most had been treated for more than 1 year. Although treatment adherence was not formally quantified, all patients were under regular follow-up at our pediatric clinic. The diagnosis of FMF was established according to the Yalçınkaya criteria [35], and *MEFV* gene mutations were assessed in the genetic analyses of the patients. Patients diagnosed with FMF were classified into mild (n = 32), moderate (n = 32), and severe (n = 32) disease groups according to the original Pras disease severity score and its established cut-off values [36]. The participant recruitment and selection process is summarized in Figure 3.

Triglyceride (TG), parathyroid hormone (PTH), alkaline phosphatase (ALP), phosphorus (P), calcium (Ca), high-density lipoprotein cholesterol (HDL-C), low-density lipoprotein cholesterol (LDL-C), total cholesterol (TC), and vitamin D (25-OH vitamin D) levels were measured in the patients, and bone mineral density (BMD) was assessed. Clinical symptoms (abdominal pain, fever, arthritis, chest pain, and similar findings) and *MEFV* gene mutations of the patients diagnosed with FMF were recorded.

Cardiovascular risk indices were calculated using established formulas reported in the literature, including the Atherogenic Index of Plasma (AIP), Atherogenic Coefficient (AC), Castelli Risk Index-I (CRI-I), and Castelli Risk Index-II (CRI-II) [9,37,38,39]. AIP was calculated as log(TG/HDL-C), AC as (TC−HDL-C)/HDL-C, CRI-I as TC/HDL-C, and CRI-II as LDL-C/HDL-C.

All patients were evaluated during attack-free (intercritical) periods. Clinical and laboratory assessments were performed when no acute FMF attack was present in order to minimize the potential confounding effects of inflammatory exacerbations on laboratory parameters. All blood analyses were performed on samples obtained after at least 10–12 h of fasting, following written informed consent at the time of admission. Serum concentrations of TG, TC, HDL-C, and LDL-C were analyzed by spectrophotometry (Beckman Coulter AU 5800, Brea, CA, USA), and Ca, P, ALP, PTH, and vitamin D concentrations were analyzed by chemiluminescence and spectrophotometry (Ca, P, and ALP: Beckman Coulter AU5800 clinical chemistry analyzer Inc., Brea, CA, USA; PTH and vitamin D: Beckman Coulter UniCel DxI 800 Access immunoassay analyzer Inc., Brea, CA, USA). Bone mineral density (BMD) measurements were performed at the lumbar vertebrae (L1–L4) using the dual-energy X-ray absorptiometry (DEXA) method (Stratos DR, DMS Imaging, Le Montueux, France), after the patient had been informed about radiation exposure and written informed consent had been obtained. The results were obtained in g/cm^2^ and recorded as standard deviation scores adjusted for age and sex (BMD SDS) using the Chıld Metrıcs calculation program developed by Ceddcozum [40]. *MEFV* gene mutations were analyzed by real-time PCR (DNA-Technology, Moscow, Russia).

The four groups were compared in terms of demographic data, AIP, AC, CRI-1, and CRI-2, as well as Ca, P, ALP, PTH, vitamin D, and BMD SDS values. Spearman correlation analysis was performed to investigate whether an association existed between BMD SDS values and cardiovascular risk indices in patients diagnosed with FMF. Patients with FMF were divided into two groups, those carrying the *M694V* mutation and those carrying mutations other than *M694V*, and they were compared with respect to AIP, AC, CRI-1, CRI-2, and BMD SDS values.

### 4.1. Exclusion Criteria

Individuals with the following conditions were excluded from the study: diabetes mellitus, chronic kidney disease, liver disease, or endocrine disorders; treatment with medications known to affect bone metabolism, including corticosteroids, antiepileptic drugs, methotrexate, and biological agents; obesity or malnutrition; and concomitant chronic inflammatory diseases, such as juvenile idiopathic arthritis, inflammatory bowel disease, systemic lupus erythematosus, and vasculitis.

### 4.2. Statistical Analysis

SPSS program, ver26.0 (IBM Corp., Armonk, NY, USA), was used to conduct statistical analyses. The Kolmogorov–Smirnov test was used to determine if continuous variables were normal. While non-normally distributed data were displayed as median (IQR), regularly distributed continuous variables were portrayed as mean ± SD. ANOVA for regularly distributed variables and the Kruskal–Wallis test for non-normally distributed variables were used to compare four separate groups. When necessary, the Tukey test was used for post hoc pairwise comparisons. Spearman’s rank correlation coefficient was used to evaluate correlations between continuous variables. The independent samples *t*-test or the Mann–Whitney U test, if applicable, were used to compare FMF patients with and without mutations. Distribution patterns were illustrated via pie charts. A two-sided method with a 95% confidence level was used for all statistical analyses. A *p*-value of less than 0.05 was deemed statistically significant.

## 5. Conclusions

In children and adolescents with FMF, cardiovascular risk and bone health should be evaluated through a holistic approach that takes disease severity and genetic features into account. The inclusion of cardiovascular risk indices in routine follow-up and the regular monitoring of bone mineral density in the severe disease group may contribute to the early recognition of possible long-term complications and to the early implementation of preventive approaches.

### 5.1. Strengths

Unlike previous studies that primarily focused on cardiovascular risk indices alone, the present study simultaneously evaluated cardiovascular risk markers, bone mineral density, disease severity, and mutation status in a pediatric FMF cohort, providing a broader perspective on the potential long-term consequences of the disease.

### 5.2. Limitations

The present study has several limitations. First, inflammatory biomarkers such as C-reactive protein, erythrocyte sedimentation rate, serum amyloid A, interleukin-1β, interleukin-6, and tumor necrosis factor-α were not systematically assessed. Since chronic inflammation is considered a major mechanism linking FMF with both increased cardiovascular risk and altered bone metabolism, the absence of these biomarkers limited our ability to directly investigate the biological pathways underlying the observed associations. Second, the cross-sectional design precludes causal inference and does not allow assessment of temporal changes in cardiovascular risk or bone mineral density. Third, the single-center design and the relatively limited sample size may restrict the generalizability of the findings.

Furthermore, potentially important confounding factors such as physical activity level, dietary habits, pubertal status, cumulative inflammatory burden, and colchicine exposure were not evaluated in detail. Future studies incorporating these variables may provide a more comprehensive understanding of the relationship between FMF severity, cardiovascular risk, and bone health.

In addition, potential confounding factors that may affect the relationship between BMD SDS and cardiovascular risk indices were not formally controlled for using multivariable statistical models. Therefore, the observed correlations should be interpreted cautiously. Furthermore, obesity, malnutrition, and other factors that may influence bone metabolism and cardiovascular risk were not evaluated as covariates. Although these conditions were excluded to reduce heterogeneity, this approach may have limited the generalizability of the findings.

A limitation of this study is that disease severity was assessed using the Pras disease severity score, which was originally developed for adult FMF populations. Therefore, the score may not fully capture disease severity characteristics specific to pediatric patients.

## Figures and Tables

**Table 1:** Comparison of cardiovascular risk indices among FMF severity groups and controls.

Parameters	Severe Group (n:32)	Moderate Group (n:32)	Mild Group (n:32)	Control (n:30)	Test Value	*p*	Post Hoc Comparison
AIP	0.36 ± 0.34	0.22 ± 0.17	−0.06 ± 0.15	−0.07 ± 0.21	27.262	<0.001	1–3, 1–4, 2–3, 2–4
AC	2.95 ± 1.15	2.13 ± 0.77	1.70 ± 0.42	1.62 ± 0.39	20.406	<0.001	1–2, 1–3, 1–4, 2–4
CRI-1	3.95 ± 1.15	3.13 ± 0.77	2.70 ± 0.42	2.62 ± 0.39	20.406	<0.001	1–2, 1–3, 1–4, 2–4
CRI-2	2.59 ± 1.03	1.99 ± 0.53	1.52 ± 0.32	1.46 ± 0.31	22.025	<0.001	1–2, 1–3, 1–4, 2–3, 2–4

AIP: Atherogenic Index of Plasma, AC: Atherogenic Coefficient, CRI-1: Castelli Risk Index-1, CRI-2: Castelli Risk Index-2.

**Table 2:** Actual lipid parameters of the groups.

Parameter	Severe Group (n = 32)	Moderate Group (n = 32)	Mild Group (n = 32)	Control (n = 30)	F	*p*
TC (mg/dL)	165.91 ± 23.37	152.41 ± 23.60	144.19 ± 32.59	162.80 ± 13.55	5.276	0.002
HDL (mg/dL)	42.50 ± 11.00	47.66 ± 7.44	52.81 ± 3.82	62.50 ± 5.01	41.206	<0.001
LDL (mg/dL)	102.97 ± 19.73	89.50 ± 16.57	84.81 ± 16.88	84.70 ± 9.52	8.945	<0.001
TG (mg/dL)	120.84 ± 78.02	75.75 ± 22.32	57.22 ± 17.21	50.50 ± 5.09	18.070	<0.001

TC: Total Cholesterol, HDL: High-Density Lipoprotein Cholesterol, LDL: Low-Density Lipoprotein Cholesterol, TG: Triglycerides.

**Table 3:** Comparison of BMD SDS values and bone metabolism parameters in FMF patients and the control group.

Parameters	Severe Group (n:32)	Moderate Group (n:32)	Mild Group (n:32)	Control (n:30)	Test Value	*p*	Post Hoc Comparison
Ca	9.40 (9.22–9.77)	9.30 (9.2–9.5)	9.35 (9.2–9.67)	9.40 (9.20–9.72)	2.112	0.550	
P	5.09 ± 0.41	5.06 ± 0.43	5.04 ± 0.59	5.07 ± 0.38	0.074	0.974	
ALP	167.66 ± 27.82	166.59 ± 15.54	165.34 ± 8.81	168.30 ± 14.21	0.161	0.922	
Vitamin D	23.0 (22.0–25.75)	24.0 (23.0–26.75)	24.0 (23.0–26.0)	24.0 (23.0–27.0)	2.476	0.480	
PTH	47.15 (47.0–48.0)	47.0 (46.25–48.0)	47.0 (46.22–48.0)	47.0 (45.0–48.0)	1.515	0.679	
BMD SDS	0.29 (−1.22–1.77)	0.90 (0.40–1.37)	0.72 (0.13–2.65)	1.43 (0.57–2.27)	9.369	0.025	1–4

Ca: Calcium, P: Phosphorus, ALP: Alkaline phosphatase, PTH: Parathyroid hormone, BMD SDS: Bone mineral density standard deviation score.

**Table 4:** Spearman correlation analysis of BMD SDS and cardiovascular risk indices.

	AIP	AC	CRI-1	CRI-2
BMD SDS	rho = −0.041 *p* = 0.647	rho = −0.117 *p* = 0.191	rho = −0.117 *p* = 0.191	rho = −0.046 *p* = 0.612
AIP		rho = 0.499 *p* < 0.001	rho = 0.499 *p* < 0.001	rho = 0.570 *p* < 0.001
AC			rho = 1.000	rho = 0.864 *p* < 0.001
CRI-1				rho = 0.864 *p* < 0.001
CRI-2				

AIP: Atherogenic Index of Plasma, AC: Atherogenic Coefficient, CRI-1: Castelli Risk Index-1, CRI-2: Castelli Risk Index-2, BMD SDS: Bone mineral density standard deviation score.

**Table 5:** Metabolic and bone-related parameters according to mutation status in FMF patients.

	Mutation Other Than M694V (n:60)	Mutation M694V (n:36)	Test Value	*p*
AIP	0.19 ± 0.28	0.17 ± 0.30	0.295	0.769
AC	2.30 ± 0.94	2.23 ± 0.99	0.312	0.756
CRI-1	3.30 ± 0.94	3.23 ± 0.99	0.312	0.756
CRI-2	1.98 ± 0.84	2.06 ± 0.79	−0.499	0.619
BMD SDS	0.38 (−1.09–1.13)	0.95 (0.15–2.67)	782.5	0.011

AIP: Atherogenic Index of Plasma, AC: Atherogenic Coefficient, CRI-1: Castelli Risk Index-1, CRI-2: Castelli Risk Index-2, BMD SDS: Bone mineral density standard deviation score.

## Data Availability

The original contributions presented in this study are included in the article. Further inquiries can be directed to the corresponding author.
